# The Burden of Adult X-Linked Hypophosphatemia on Carers and Family Members: A Mixed-Methods Study

**DOI:** 10.36469/001c.133860

**Published:** 2025-04-24

**Authors:** Elina Matter, Claire Lawrence, Oliver Gardiner, Victoria Hayes, Gillian Logan, Ben Johnson, Andrew Lloyd

**Affiliations:** 1 Acaster Lloyd Consulting Ltd., London, UK; 2 XLH UK, Kington, UK; 3 Kyowa Kirin International, plc, Marlow, UK

**Keywords:** health-related quality of life, spillover effects, EQ-5D-5L, Work Productivity and Activity Impairment Questionnaire, health technology assessments, X-linked hypophosphatemia

## Abstract

**Introduction:** X-linked hypophosphatemia (XLH) is a rare, genetic disorder that severely impacts the health-related quality of life (HRQoL) of people living with the condition. This impact can also extend to carers and family members, described as a “spillover effect.” Measurement of spillover effects can lead to greater understanding of disease burden and potentially to more equitable decision-making regarding adoption of treatments by health technology assessment (HTA) bodies. This study aimed to explore spillover effects among carers and family members of adults with XLH. **Methods:** This cross-sectional, mixed-methods study included carers and family members of adults diagnosed with XLH in the United Kingdom. Quantitative data included a background questionnaire, the EQ-5D-5L, and the Work Productivity and Activity Impairment Questionnaire (WPAI). Qualitative data were gathered through semi-structured interviews. **Results:** Twenty carers or family members of adults with XLH completed the study. Four (20%) had an XLH diagnosis themselves. Mean (SD) EQ-5D utility was 0.66 (0.33) for the total sample, and 0.20 (0.31) and 0.77 (0.21) for participants with and without a personal diagnosis of XLH, respectively. The WPAI activity impairment was 42% and overall work impairment was 28%. Interviews revealed 6 areas of participants’ lives impacted by XLH: emotional wellbeing, daily activities, work, finances, social lives and relationships, and physical impacts. **Conclusion:** Findings of this study indicate a substantial health-related quality-of-life and productivity burden for carers and family members of adults with XLH. The magnitude of this burden highlights the importance of incorporating such spillover effects in HTAs and broader rare disease policies.

## INTRODUCTION

X-linked hypophosphatemia (XLH) is a rare, genetic, phosphate-wasting disorder caused by loss-of-function mutations in the Phosphate Regulating Endopeptidase Homolog X-Linked (*PHEX*) gene.^1^ These mutations result in increased production of FGF23, leading to chronic hypophosphatemia and reduced synthesis of active vitamin D.^2^ The global prevalence of XLH is estimated to range from 1.4 to 4.8 per 100 000 individuals.^3^ XLH usually manifests in childhood with the presentation of bowed legs, with symptoms progressing into adulthood.^4–6^ Adults with XLH have an increased risk of fractures and pseudofractures, osteoarthritis, and other musculoskeletal symptoms, with a consequent impact on health-related quality of life (HRQoL), including an impact on mobility, daily function, and mental wellbeing.^1,7–9^

Pharmacological treatment of XLH has historically consisted of supplementation with oral phosphate and active vitamin D, with the goal of improving serum phosphate levels to ameliorate symptoms. However, this treatment does not address the fundamental cause of XLH.^10,11^ In recent years, burosumab, a monoclonal antibody that targets FGF23, has been approved as a treatment option for XLH.^12,13^ Burosumab has been shown in clinical trials to improve serum phosphate concentration, osteomalacia, fracture healing, and symptoms in adults with XLH.^14,15^

The impact of XLH can extend beyond the affected individual to family members due to the psychosocial impact or the need to provide informal care and support for an individual.^16^ This negative impact has been described as a spillover effect.^17–20^ For rare diseases, spillover effects may be greater due to poor understanding of the condition, lack of support, and limited treatment options.^20,21^ Carers or family members of individuals with inherited diseases, such as XLH, may also be diagnosed with the condition, which may lead to a greater burden.^21–23^

Measurement of spillover effects can enable a better understanding of disease burden and the extent to which it extends beyond the affected individual.^18,24^ Incorporating these broader effects in decision models may produce more equitable decision-making regarding the adoption of treatments. The inclusion of spillover effects in economic evaluations of interventions is accepted under certain circumstances by some health technology assessment (HTA) agencies.^24,25^ For example, the National Institute for Health and Care Excellence (NICE) states that economic evaluations should incorporate health effects for carers where relevant.^26^

Although spillover effects are typically measured quantitatively, leveraging both quantitative and qualitative methods provide a fuller picture of disease burden among carers and family members. Established quantitative measures of HRQoL and productivity/activity impairment can provide a general understanding of the impact of spillover effects. Qualitative methods produce rich, first-hand accounts that provide in-depth understanding of disease burden and potential explanations for quantitative findings.^27^

Limited evidence exists on spillover effects on broader family networks of individuals with musculoskeletal conditions, as highlighted in a recent targeted literature review.^28^ Previous research in XLH focused on exploring the burden for carers of children and adolescents with XLH^23^ or focused primarily on carers’ needs rather than their lived experiences.^21^

The current study used a mixed-methods approach to explore informal carers’ and family members’ experiences living with, caring for, or supporting an adult with XLH.

## METHODS

### Study Design and Participants

This cross-sectional study employed a mixed-methods survey approach with informal carers and family members of adults diagnosed with XLH in the United Kingdom (UK). Participants were recruited through XLH UK,^29^ the only patient group registered in the UK to support families and individuals with XLH. Recruitment took place between February 2023 and April 2023 via advertisements shared on social media groups by XLH UK for individuals diagnosed with XLH. To participate in the study, individuals had to be either an unpaid informal carer or family member (aged ≥18 years) of an adult diagnosed with XLH. They also had to be living in the UK and able and willing to provide informed consent to complete a questionnaire and participate in a one-to-one virtual interview.

Interested participants were given a study link to access the online survey platform Qualtrics. The platform included a brief screener questionnaire to assess participants’ eligibility. Eligible participants were guided to the participant information sheet, followed by an online consent form. Participants who consented were referred to a background questionnaire to complete online and were invited to participate in a semistructured one-to-one interview. Interviews took place over the telephone or via Zoom. At the start of each interview, participants reconfirmed their consent verbally. All interviews were audio-recorded, transcribed verbatim, and de-identified for analysis purposes.

### Quantitative Outcomes: Health-Related Quality of Life and Work Productivity

Quantitative outcomes collected through the background questionnaire included participants’ demographic data, XLH status, relationship to the care recipient, and clinical information relating to themselves and their care recipients. Participants’ HRQoL was assessed using the EQ-5D-5L.^30^ EQ-5D-5L data were summarized as domain-level responses, utility values, and EQ visual analog scale (VAS) responses. Utilities were estimated based on UK preference weights.^31^ Impairment in work and activities was assessed using the Work Productivity and Activity Impairment Questionnaire (WPAI), an instrument designed to measure impairment in paid work and regular daily activities.^32^

### Qualitative Outcomes: Participant Interviews

A bespoke semistructured interview guide was developed with input from XLH UK, who provided guidance on the topics for inclusion and reviewed the wording of interview questions. The interview guide included open-ended questions covering the participants’ experience of caring for, living with or supporting an adult with XLH. Topics included their role and types of support provided, their role supporting the management of the condition of the adult with XLH, and impacts they experienced due to caring for, living with, or supporting an adult with XLH. For carers with an XLH diagnosis, additional questions explored how their own condition affected their caring role, including any unique challenges or benefits of sharing the same diagnosis as the person they care for.

### Data Analysis

Analyses were conducted for the total sample and subsamples based on participants’ XLH status. Sociodemographic and clinical data were summarized descriptively. Categorical variables were summarized as counts and percentages. Continuous variables were summarized as means, SD, and ranges. The EQ-5D-5L data were mapped to the EQ-5D-3L using the mapping function by Hernández-Alava et al to generate utility weights.^31^ Individual participants’ EQ-5D utilities were compared with expected utilities based on age-stratified EQ-5D-3L reference values for the UK general population.^33^ Mean differences (mean individual observed utility minus mean individual expected utility) were also calculated. The WPAI’s 4 domain scores (absenteeism, presenteeism, work productivity loss, and activity impairment) were calculated using developer’s guidelines.^34^

The interview transcripts were analyzed using content analysis.^35^ A coding framework was developed based on the questions asked and information reported in the interviews. Two interviewers independently coded a selection of interview transcripts. Following this, a comparison and reconciliation process occurred in which all codes were compared, discussed, and reconciled wherever differences occurred. Once sufficient agreement between the coders was established, the framework was updated and the remaining transcripts were coded. The data were reviewed and responses to questions were summarized and illustrated with quotes. The qualitative data analysis was conducted using MAXQDA,^36^ a software tool designed to organize qualitative and mixed-methods data. Experienced qualitative researchers conducted the analysis.

## RESULTS

### Participant Characteristics

Twenty informal carers or family members of adults with XLH, who expressed interest in participation, were screened, and met inclusion criteria were included in the survey (**Table 1**). Most participants providing care or support for an adult with XLH were male (65%) and currently employed (70%).

**Table 1.** Sociodemographic Characteristics of Carers and Family Members

	**Total Sample (N = 20)**	**Participants With XLH (n = 4)**	**Participants Without XLH (n = 16)**
Age, y			
Mean (SD)	48.6 (17.0)	45.3 (12.5)	49.4 (18.2)
Range	26-83	29-59	26-83
Gender, n (%)			
Female	7 (35)	3 (75)	4 (25)
Male	13 (65)	1 (25)	12 (75)
Current living situation, n (%)			
Living with partner/spouse	14 (70)	1 (25)	13 (81)
Living alone	2 (10)	1 (25)	1 (6)
Living with relative(s)	4 (20)	2 (50)	2 (13)
Current employment status, n (%)			
Employed, full-time	10 (50)	1 (25)	9 (56)
Employed, part-time	4 (20)	2 (50)	2 (13)
Unable to work due to health issues	1 (5)	1 (25)	—
Retired	3 (15)	—	3 (19)
Other	2 (10)	—	2 (13)
Highest level of educational attainment, n (%)			
Secondary education	8 (40)	2 (50)	6 (38)
Degree level or higher	10 (50)	2 (50)	8 (50)
Other	2 (10)	—	2 (13)
Receiving social security benefit payments, n (%)			
Yes	7 (35)	3 (75)	4 (25)
No	12 (60)	—	12 (75)
Prefer not to answer	1 (5)	1 (25)	—
Carer or family member relation to individual with XLH, n (%)			
Parent	9^a^ (35)	5b (83)	4c (20)
Child	2^a^ (8)	1b (17)	1c (5)
Sibling	2^a^ (8)	—	2c (10)
Partner/spouse	10^a^ (39)	—	10c (50)
Grandparent	2^a^ (8)	—	2c (10)
Father-in-law	1^a^ (4)	—	1c (5)
Living in same household with individual with XLH, n (%)			
Yes	20^a^ (77)	5 ^b^ (83)	15^c^ (75)
Other family members with XLH diagnosis,^d^ n (%)			
Mother	1 (5)	1 (25)	
Child	7 (35)	—	7 (44)
Brother or sister	1 (5)	1 (25)	—
Grandmother or grandfather	1 (5)	1 (25)	—
Niece or nephew	2 (10)	—	2 (13)
Other	2 (10)	—	2 (13)
Other illnesses/conditions, n (%)			
Yes	8 (40)	3 (75)	5 (31)
No	12 (60)	1 (25)	11 (69)

Abbreviation: XLH, X-linked hypophosphatemia.

^a^Sum of counts includes N = 26 adults with XLH being cared for by participants.

^b^Sum of counts includes N = 6 adults with XLH being cared for by participants with an XLH diagnosis.

^c^Sum of counts includes N = 20 participants being cared for by participants without an XLH diagnosis.

^d^Family members with XLH other than those being reported on in the study who were not adults with XLH (aged <18 years old) or for which participants were not living with or providing care/support for.

Overall, the 20 carers and family members reported data on 21 separate adults with XLH. Participants were allowed to describe caring for more than 1 adult with XLH; the sample included 5 participants caring for the same adult. This resulted in 26 reports of caring for, supporting, or living with an adult with XLH. Participants were most commonly a partner or spouse (39%) or a parent of an adult with XLH (35%).

Four participants reported an XLH diagnosis themselves (20%). **Table 2** presents the treatment history of carers with an XLH diagnosis and care recipients. Oral phosphate and vitamin D (eg, alfacalcidol or calcitriol) supplements were the most reported treatments received by both care recipients (90% and 86%, respectively) and participants with XLH (100%).

**Table 2.** Treatment History of Participants With XLH and Care Recipients

**Treatment**	**Participants With XLH (N = 4)**	**Care Recipients (N = 21)^a^**
Oral phosphate supplements		
Currently receiving	1 (25%)	4 (19%)
Length of treatment (y), mean (SD)	47.0^b^	16.5 (13.0)
Ever received	4 (100%)	18 (86%)
Vitamin D (eg, alfacalcidol or calcitriol)		
Currently receiving	2 (50%)	5 (24%)
Length of treatment (y), mean (SD)	36.0 (15.6)	14.7 (13.4)
Ever received	4 (100%)	18 (86%)
Burosumab		
Currently receiving	2 (50%)	8 (38%)
Length of treatment (y), mean (SD)	2.0 (0.0)	3.0 (1.6)
Ever received	2 (50%)	10 (48%)

^a^Sum of counts does not equal N=26 because for some care recipients more than 1 carer participated in the study; therefore, the care recipient was counted once.

^b^Value represents total length of treatment; mean (SD) was not calculated because only 1 individual reported currently receiving oral phosphate supplements.

### Health-Related Quality-of-Life and Work Productivity Data

**EQ-5D:** The mean EQ-5D utility for the total sample was 0.66 (SD = 0.33). Mean EQ-5D utilities for participants with and without a personal diagnosis of XLH were 0.20 (SD = 0.31) and 0.77 (SD = 0.21), respectively. EQ-5D-5L dimension level data are in the **Supplementary Material**. Participants reported some degree of problems in mobility (50%), pain and discomfort (55%), usual activities (45%), and anxiety and depression (65%) dimensions. The frequency of problems reported on EQ-5D dimensions was substantially higher in participants with XLH.

**Figure 1.**
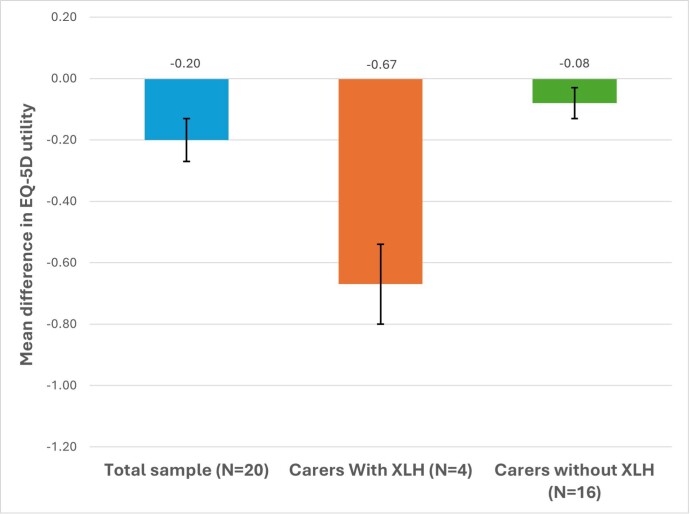
Mean Differences (SE) in EQ-5D Utility for Study Participants Compared With the UK General Population Abbreviations: XLH, X-linked hypophosphatemia; SE, standard error.

Overall, the sample had a lower health-related quality of life (HRQoL) than the UK general population, based on age-matched mean differences (**Figure 1**).

The mean EQ-VAS score for the total sample was 65.3 (SD = 25.6). Mean EQ-VAS scores for participants with a personal diagnosis of XLH were substantially lower (26.0; SD = 10.9) than participants without a personal diagnosis of XLH (75.1; SD = 17.1).

**Figure 2.**
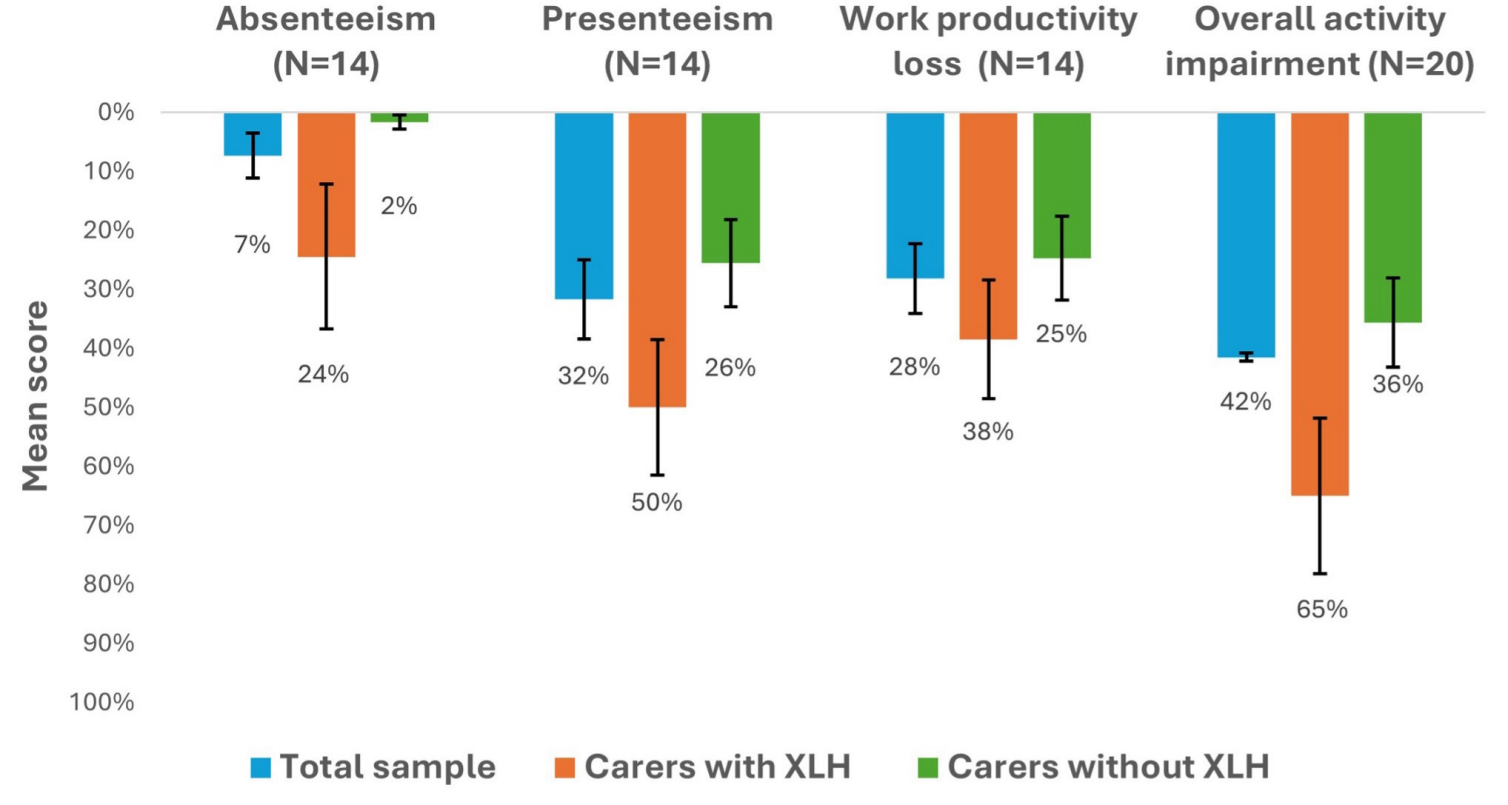
Participant Absenteeism, Presenteeism, Work Productivity Loss, and Activity Impairment Abbreviations: XLH, X-linked hypophosphatemia; SE, standard error. Note: High scores indicate greater impairment and lower productivity.

**WPAI**: Most participants were employed (14/20; 70%) and had worked in the past 7 days (12/14; 86%). The mean percentage work productivity loss was 28%. Across the total sample, the overall activity impairment was 42%, with participants with XLH showing greater work and productivity impairment across all domains. **Figure 2** presents participants’ mean reported absenteeism, presenteeism, work productivity loss, and activity impairment.

### Interview Findings

**Figure 3.**
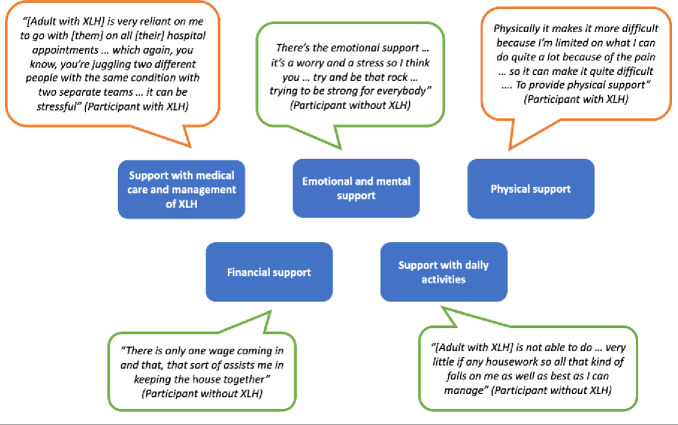
Key Areas of Support Described by Carers and Family Members of Adults With XLH

**Support provided by carers and family members**: The qualitative interviews revealed 5 areas where carers or family members of adults with XLH provide support. Participants described spending significant time providing support, ranging from a “couple of hours a day” to “15 hours a week.” For some participants, this involved using all their “spare time” to provide support, with 1 participant describing it as “another work shift.” Fractures, pseudofractures, and disease progression were all factors reported by carers and family members that increased the support provided. An overview of areas in which carers or family members provide support is presented in **Figure 3** alongside selected illustrative quotes.

Quotations bordered in orange were from participants with an XLH diagnosis; those bordered in green were from participants without an XLH diagnosis.

- *Support with medical care and management of XLH:* All participants had a role in the medical care and management of XLH for the individual for whom they provide care or support. This included support with medication, providing transport to medical appointments, assisting with pain management, and providing support after medical procedures. One participant with XLH reported that providing this type of support was difficult due to their own diagnosis.
- *Physical support*: All but 2 participants reported providing physical support for the individual with XLH. For some, this involved support with mobility, while for others, it also meant support with self-care. The 4 participants who had an XLH diagnosis themselves reported that their own XLH diagnosis had made it more challenging to provide physical support.
- *Emotional and psychological support:* Emotional and mental support included providing comfort, reassurance, and a “listening ear,” with 1 participant noting that they provide “emotional support 24 hours a day” [participant with XLH].
- *Support with daily activities:* Support with daily activities involved supporting care recipients with cooking, housework, shopping and childcare. For one participant with an XLH diagnosis themselves, having to take on more responsibility with housework was described as putting added “pressure” on them.
- *Financial support:* Participants described several ways in which they provide financial support, such as paying for 2 cars due to the care recipient being unable to get around without a car or having to pay for a cleaner each week. One participant with an XLH diagnosis indicated that they struggle to provide financial support due to their own condition preventing them from working.

**Figure 4.**
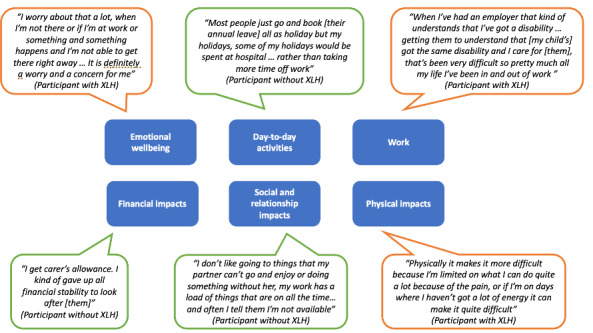
Key Impacts Described by Carers and Family Members of Adults With XLH

**Impact on carers and family members**: Carers and family members described impacts to 6 key areas of their lives resulting from living with, caring for, or providing support for an adult with XLH. An overview of impacts is provided in **Figure 4** with illustrative quotes.

Quotations bordered in orange were from participants with an XLH diagnosis; those bordered in green were from participants without an XLH diagnosis.

- *Emotional wellbeing*: All but 1 participant reported a negative impact on their emotional well-being (including worry, stress, and guilt) associated with caring for or living with an adult with XLH. Carers and family members worried about disease progression of the adult with XLH and their inability to cope without support. They also worried about their own ability to continue providing support.

*“Sometimes I feel like I have to try much harder to provide the support that I do. And sometimes maybe it stretches me a little bit far, and I can’t help but feel guilty about that.”* [Participant without XLH]

Participants with XLH themselves also experienced guilt associated with passing on the condition as well as experiencing the added burden of worrying about their own health.

“*I can hardly look after myself, so yeah it does have an impact … But then I’ve just got to find an extra little thing to you know drive up to [location] or wherever and go to an appointment with [adult with XLH]*.” [Participant with XLH]

- *Day-to-day activities:* Providing care for an adult with XLH was reported as having an impact on participants’ daily activities, including having less time for themselves or less freedom to engage in activities they would have otherwise liked to engage with. Some participants noted that this was due to planning their day around the care recipient’s support needs. For others, this was also associated with worry about leaving them alone for too long.

“*I’m limited basically, I work and I care and that’s about the limitation to my life. I can’t say that I’ve got any outside activities because I haven’t, I’ve got no energy for them after I’ve finished doing what I need to do. I wish I could say more, I just don’t have a life outside of XLH, my life is consumed by it*.” [Participant with XLH]

- *Work*. Providing care or support affected respondents’ ability to work in several ways. Some respondents reported taking time off to accompany their partner or family members with XLH to medical appointments. Others described working in ways they would have otherwise not chosen to meet their care recipient’s support needs. Some participants also reported having had to stop working altogether to be able to provide care or support.

“*I mean to be honest I pretty much stopped work when I became a carer for [Adult with XLH]. As I say, I’d kind of pretty much quit working and went on Carer’s Allowance.*” [Participant without XLH]

- *Financial impact.* The majority of participants reported a financial impact. For most, this was associated with managing XLH, including dental treatment costs, paying for prescriptions, and home adaptations made to meet care recipients’ support needs. Other contributing factors included transport costs and taking time off work to provide support.

“*I was at one time drawing out say £5000 and giving it to one of them for their teeth and the other for other things and that was dripping away*.” [Participant without XLH]

- *Social and relationship impact.* Participants also described limitations in their ability to socialize with friends and other family members due to factors associated with supporting an adult with XLH. This included limited social outings due to the pain or accessibility issues experienced by the care recipient. As a result, participants described prioritizing their care recipient’s support needs over social activities. Due to limited available time, carers and family members also reported a negative impact on relationships. However, positive impacts were also described, such as strengthening relationships and finding new ways of spending quality time together. For participants with XLH, the shared experience of having an XLH diagnosis was also noted to have a positive impact on their relationship with the care recipient.

“*It’s almost brought us a bit closer really because it’s something we share in common, and we talk about and not a lot of other people understand very well. So through supporting each other, I guess it’s brought us closer*.” [Participant with XLH]

- *Physical impact.* Physical burden was also noted. Participants described experiencing sleeplessness, tiredness, and body aches associated with the physical demands of providing care. For carers living with XLH, the physical impact reported was exacerbated by their own diagnosis.

*” The most challenging thing that I have to battle with is pain, working through my own pain whilst caring for somebody else, it can be so challenging, I can’t even put it into words actually, I just want it to stop.”* [Participant with XLH]

- *Change over time in carer impact.* Participants described changes in the burden of care due to the adult with XLH receiving novel treatments. Improved outcomes for patients were reportedly associated with a reduced burden for carers. This included a reduction in the amount of support provided, taking less time off work to provide support, and improvements in their own emotional wellbeing due to reduced worry.

*“It’s changed dramatically [time spent providing support]. If I’m working late then I come home and [Adult with XLH] is doing the dinner. Weekends we share the work with shopping and housework. Gardening, we can both do together now. Walking the dog, we can both do together now as well.”* [Participant without XLH]

Two participants also reported an increased impact of care related to the care recipient receiving the novel treatment. One related to a change in treatment administration procedures. The other related to increased emotional support provided due to the care recipient experiencing an increase in bone pain and restlessness following their treatment.

## DISCUSSION

This mixed-methods study is the first to explore spillover effects of XLH on carers and family members of adults with XLH. Quantitative results indicated impacts on HRQoL and productivity, which were greater for people who also had XLH themselves. Qualitative findings revealed substantial impacts on several areas of carers’ and family members’ lives. These findings add to the limited research in musculoskeletal conditions and rare diseases, aligning with a qualitative study with parents of adolescents with XLH,^23^ suggesting that similar challenges persist across age groups. The spillover effects observed indicate that the broader family impact of XLH affects caregivers and family members of adults, reinforcing the condition’s widespread burden.

The study highlights the unique burden experienced by carers who also have XLH. The mean EQ-5D score for these individuals (0.20) was substantially lower than that reported by patients with XLH in other studies conducted in the UK (0.65) and Spain (0.56).^37,38^ Loss of work productivity for carers with XLH was much higher (compared with those without XLH), with higher presenteeism and absenteeism.

The study’s qualitative findings revealed areas of carer burden not fully captured by quantitative measures. Most carers without XLH reported “No problems” with their usual activities on the EQ-5D (11/16; 69%), yet almost all participants qualitatively described substantial impacts on their day-to-day activities. Similarly, all but 1 participant described impacts on their emotional wellbeing, while most without XLH reported either “No problems” (7/16; 44%) or only “Slight problems” (5/16; 31%). These discrepancies indicate that the EQ-5D may not adequately capture all aspects of carer and family member burden. This finding is supported by previous research in carers of patients with other chronic diseases.^39^

The extensive burden identified highlights the need to support the integration of spillover effects in HTAs. Currently, spillover effects are not routinely considered: a review of NICE appraisals found that caregiver HRQoL was only included in 12 of 414 technology appraisals and 4 of 8 Highly Specialised Technology appraisals.^24^ The former included 1 appraisal for a rare disease, while the latter were all in rare diseases, including 1 in children and young people with XLH, in which the NICE committee noted the importance of considering carer burden in its assessment.^40^

Our findings suggest that novel treatments for XLH patients may positively affect carer burden, with some participants describing decreased support needs and improved emotional wellbeing.

This study’s mixed-methods approach demonstrates the value of collecting both quantitative and qualitative data when measuring spillover effects. Given the limitations of the EQ-5D, novel validated measures and a framework for qualitative evidence on spillover effects should be developed. The findings also highlight the need to consider carers and family members in service delivery and recognize conditions affecting multiple family members across generations may have higher unmet needs. Likewise, routinely including spillover effects in cost-utility analysis in HTA could change the landscape of the approval process for new treatments, potentially leading to changes in drug development priorities.

There are some limitations of the study. First, some participants referred to the same adult diagnosed with XLH in their interview. Best efforts were made to ensure that patients were not counted more than once when summarizing patient characteristics. However, it is possible that some connections between adults with XLH and carers were not disclosed. Second, the limited number of carers who also had XLH introduced a degree of uncertainty in the findings for this subset of respondents. As such, the interactions between being a patient and a carer were difficult to fully conceptualize and may not have been adequately captured. Thirdly, while this study focused on the immediate impacts on carers and family members, spillover effects may extend to wider social networks, such as friends, which were not explored in this study. Finally, as the EQ-5D only measures HRQoL, other aspects of quality of life may have been overlooked in the quantitative analysis, although these aspects were captured in qualitative interviews.

Future research surveying individuals within a patient’s broader network, such as nonfamily household members, may be beneficial in further conceptualizing spillover effects in XLH. In addition, research in other inherited or genetic conditions would be beneficial in understanding the unmet needs of carers affected by the condition themselves.

In conclusion, this study highlights that the impact of XLH in adults extends beyond the patient, exerting a substantial quality of life and productivity burden on carers and family members.

### Data Availability

The data set generated and analyzed during the current study is not publicly available to maintain the anonymity of research participants. Additional data may be available from the corresponding author on reasonable request.

### Ethics Approval

The study was reviewed by Western Institutional Review Board (WIRB-Copernicus Group Independent Review Board), and an exemption determination was provided.

### Patient Consent

Participants provided informed consent online prior to participation.

## Supplementary Material

Online Supplementary Material
