# Supplementary material for: The Burden of Adult X-Linked Hypophosphatemia on Carers and Family Members: A Mixed-Methods Study
Source: J Health Econ Outcomes Res. 2025 Apr 24;12(1):162–70. doi: 10.36469/001c.133860 (PMC12033010; doi:10.36469/001c.133860)
Supplement: Online Supplementary Material [file jheor_2025_12_1_133860_280291.pdf]

JHEOR

Journal of Health Economics and Outcomes Research

Online Supplementary Material

The Burden of Adult X-linked Hypophosphatemia on Carers and Family Members: A Mixed-Methods Study.
JHEOR. 2025;12(1):162-170. doi:10.36469/jheor.2025.133929

Table S1: Domain Level EQ-5D-5L Participant Responses

This supplementary material has been provided by the authors to give readers additional information about their work.

This is an open-access article distributed under the terms of the Creative Commons Attribution 4.0 International License (CCBY-4.0). View this license's legal deed at http://creativecommons.org/licenses/by/4.0 and legal code at http://creativecommons.org/licenses/by/4.0/legalcode for more information.

Table S1. Domain Level EQ-5D-5L Participant Responses

EQ-5D Domain	Total Sample (N=20)	Subgroups	
		Carers With XLH Diagnosis (N=4)	Carers Without XLH Diagnosis (N=16)
Mobility			
No problems	10 (50%)	–	10 (50%)
Slight problems	4 (20%)	–	4 (25%)
Moderate problems	3 (15%)	2 (50%)	1 (6%)
Severe problems	3 (15%)	2 (50%)	1 (6%)
Unable to walk about	–	–	–
Self-care			
No problems	15 (75%)	–	15 (94%)
Slight problems	3 (15%)	2 (50%)	1 (6%)
Moderate problems	1 (5%)	1 (25%)	–
Severe problems	1 (5%)	1 (25%)	–
Unable to wash or dress	–	–	–
Usual activities			
No problems	11 (55%)	–	11 (69%)
Slight problems	4 (20%)	1 (25%)	3 (19%)
Moderate problems	2 (10%)	1 (25%)	1 (6%)
Severe problems	2 (10%)	1 (25%)	1 (6%)
Unable to do usual activities	1 (5%)	1 (25%)	–
Pain/discomfort			
No pain or discomfort	9 (45%)	–	9 (56%)
Slight pain or discomfort	3 (15%)	–	3 (19%)
Moderate pain or discomfort	4 (20%)	1 (25%)	3 (19%)
Severe pain or discomfort	3 (15%)	2 (50%)	1 (6%)
Extreme pain or discomfort	1 (5%)	1 (25%)	
Anxiety/depression			
Not anxious or depressed	7 (35%)	–	7 (44%)
Slightly anxious or depressed	7 (35%)	2 (50%)	5 (31%)
Moderately anxious or depressed	4 (20%)	1 (25%)	3 (19%)
Severely anxious or depressed	2 (10%)	1 (25%)	1 (6%)
Extremely anxious or depressed	–	–	–

Abbreviations: N, number of participants; XLH, X-linked hypophosphatemia.
